# Diagnostic yield of genome sequencing for children with intellectual disability and developmental delay

**DOI:** 10.3389/fneur.2026.1966246

**Published:** 2026-09-24

**Authors:** Robert Rigobello, Morgan Driver, Laurie A. Robak, Daryl A. Scott, Liesbeth Vossaert, Jason Chibuk, Linyan Meng, Fan Xia, Christine M. Eng, Petros Giannikopoulos, Xiaonan Zhao

**Affiliations:** 1 Baylor Genetics, Houston, TX, United States; 2 Department of Molecular and Human Genetics, Baylor College of Medicine, Houston, TX, United States; 3 Texas Children’s Hospital, Houston, TX, United States; 4 Innovative Genomics Institute, Berkeley, CA, United States

**Keywords:** developmental delay, genome sequencing, intellectual disability, neurodevelopmental genetics, rare disease

## Abstract

**Background:**

Genome sequencing (GS) has recently been considered as a first-tier diagnostic tool for individuals with intellectual disability (ID) and developmental delay (DD). In this study, we assess the diagnostic yield of GS for a large cohort of children with ID/DD and describe variants contributing to genetic diagnoses in this cohort.

**Methods:**

A retrospective analysis of consecutive GS results for individuals under 18 years of age with a clinical indication of ID/DD was conducted using data from a clinical diagnostic laboratory. The present analysis included 666 individuals.

**Results:**

In this cohort, 31.1% (207/666) of children received positive GS results. There were 186 children who received a genetic diagnosis that was at least partially associated with their history of ID/DD, leading to a phenotype-specific diagnostic yield of GS for ID/DD of 27.9%. For individuals without known prior genetic testing, 35.3% (103/292) had positive GS results. There were 259 variants that led to genetic diagnoses in this cohort. Of these, 7.7% (20/259) were variants with limited to no coverage on exome sequencing.

**Conclusion:**

GS identified at least one genetic diagnosis associated with ID/DD in 27.9% of children with ID/DD as a clinical indication. An additional 3.2% of children received positive GS results associated with other features of their clinical indication. Almost 8% of variants related to the genetic diagnoses in this cohort may not have been detected through other standalone genomic technologies. These findings further support the recommendation by medical and professional societies of GS as a first-tier diagnostic test for individuals with ID/DD.

## 1 Introduction

Neurodevelopmental disorders (NDDs) are a heterogenous group of disorders which include intellectual disability (ID) and developmental delay (DD). The estimated prevalence of NDDs in the United States is 1–3% (1). The etiology of NDDs is broad, including certain prenatal exposures, perinatal or neonatal trauma or events, and genetic conditions, with genetic causes accounting for up to 50% of cases with an identified etiology (2).

The diagnostic odyssey for individuals and families affected by NDDs typically takes years and may involve many different types of diagnostic tests (3–5). One study reported that the diagnostic trajectory of individuals with ID included an average of 61 appointments with healthcare providers, 16 imaging studies, and seven genetic tests across an average of 6.6 years (5). For individuals with NDDs who have eventually been diagnosed with a genetic condition, the time between first concern of symptoms and genetic diagnosis has been shown to range from six to 138 months, with an average of 40 months (4). Delayed genetic diagnosis can lead to missed early interventions, disease progression, ineffective management, and increased emotional and financial burden (6, 7).

Previously, the recommended approach to genetic testing for individuals with ID/DD included chromosomal microarray analysis (CMA), Fragile X repeat expansion analysis, and targeted gene panels (8). However, many individuals did not receive a genetic diagnosis following this multi-step approach. Meta-analyses have shown that more comprehensive genetic tests, such as exome sequencing (ES), have higher diagnostic yields when compared to CMA or gene panels (9–11). As a result, ES and genome sequencing (GS) are now being recommended as the first-tier approach based on higher diagnostic yields, their ability to shorten diagnostic odysseys, change medical management, and improve genetic counseling. Both the American College of Medical Genetics and Genomics (ACMG) and American Academy of Pediatrics (AAP) recommend ES and GS as a first- or second-tier diagnostic test for children with ID/DD (12, 13).

GS is predicted to increase the diagnostic yield over ES as it includes a more comprehensive analysis across the genome that allows for the identification of deleterious variants in intronic and noncoding regions, mitochondrial genome variants, alterations in tandem repeats, and copy number variants (CNVs). Several studies have assessed the diagnostic yield of GS for individuals with various NDDs with yields ranging from 21–41% (14–18). However, limitations of these studies included smaller sample sizes which narrowed the breadth and diversity in type of variants identified in these studies (16, 17). Therefore, there is limited information on the additional utility of GS for individuals with ID/DD. In this study, we aim to assess the diagnostic yield of GS for a large cohort of children presenting with ID/DD and describe the types of variants contributing to the genetic diagnoses.

## 2 Materials and methods

### 2.1 Cases

A retrospective analysis was performed to examine the clinical and genetic findings from consecutive individuals under 18 years of age tested by GS at Baylor Genetics, which is a clinical diagnostic laboratory accredited by the College of American Pathologists and certified by the US Department of Health and Human Services Clinical Laboratory Improvement Amendments. The study cohort included cases with GS ordered between January 2023 and October 2024. Cases were queried for clinical indications and Human Phenotype Ontology (HPO) term labels that included the phrase “intellectual disability” or “developmental delay” in order to capture cases that contained severity descriptors when applicable. Clinical indications and HPO terms were derived from laboratory test requisition forms and medical records submitted with the test order. No phenotypic exclusion criteria were applied during case selection. For each case meeting phenotypic criteria, the most recent GS report was reviewed for test type (standard or rapid; proband, duo, or trio), overall result (positive, indeterminate, or negative), gene(s) and variant(s) identified, and inheritance information. For positive GS reports, disease descriptions were reviewed to determine if the condition was associated with any NDD phenotype. Clinical notes submitted with the test order were also reviewed to assess whether each individual had previous genetic testing. Based on clinical indications, individuals were classified as having either isolated NDDs or non-isolated NDDs, depending on the presence of phenotypic findings related to other organ systems. Isolated dysmorphic features and common pediatric conditions, such as constipation, were not factored into this determination. This study was conducted under the approval of the Baylor College of Medicine Institutional Review Board (H-41191) and in accordance with the Declaration of Helsinki. Patient consent was waived due to the use of de-identified and aggregate data per the Institutional Review Board protocol.

### 2.2 Genome sequencing

DNA was extracted from either submitted blood or buccal swab samples using standardized protocols. GS libraries were PCR-free and sequenced at 40X coverage with 2 × 150 bp reads on an Illumina NovaSeq platform. FASTQ files were processed locally using the Illumina DRAGEN pipeline (v4.2.4), aligning to GRCh38 (hg38), to call single nucleotide variants (SNVs), CNVs, regions of homozygosity, and uniparental disomy (UPD). Variants were then annotated and prioritized based on factors such as population frequency, predicted functional impact, and curated clinical significance. An internal laboratory classification system, which was based on the ACMG and Association for Molecular Pathology (AMP) guidelines, was used for variant classification. Orthogonal confirmation testing was completed for all cases with reported STR expansions. Targeted reflex RNA sequencing was performed in cases with eligible variants. RNA was extracted from peripheral blood using standard protocols and performed using an amplicon-based next-generation sequencing approach, following validated standard operating procedures.

The overall call of each GS report was based on multiple factors, including variant classification, disease inheritance pattern, and clinical phenotype. A positive report was issued when an identified variant(s) was known or likely known to be associated with a disease that was at least partially consistent with the clinical phenotype (i.e., at least one component of the phenotype was explained by the result). Positive reports could have included a pathogenic (P) or likely pathogenic (LP) variant in a gene associated with a dominant condition, two P/LP variants in a gene associated with a recessive condition, or one LP/P variant and one variant of uncertain significance favoring pathogenic (VUSP) in a gene associated with a recessive condition in which variants are assumed or known to be compound heterozygous. An indeterminate report was issued when a detected variant(s) had uncertain pathogenicity, but the associated disease was relevant to the clinical phenotype. Indeterminate reports could have included a VUSP in a gene associated with a dominant condition or two VUSP in a gene associated with a recessive condition. A negative report was issued when there were no detected variants of known or uncertain clinical significance that could be related to the clinical phenotype. Secondary findings, incidental findings, and findings in genes with no known disease association did not influence the overall call of the report, as these findings were not related to the clinical indication.

### 2.3 Data analysis

Frequencies and proportions of demographic and clinical characteristics, overall call of GS results, and variant characteristics were calculated. Chi-square tests were used to identify statistically significant differences in the proportion of cases with positive GS results across various clinical and demographic variables. An additional chi-square test was used to compare overall GS results across test type. A *p*-value cutoff of .05 was used to determine statistical significance.

## 3 Results

### 3.1 Cohort characteristics

In total, 666 individuals under 18 years of age with ID/DD were included in the present analysis. Demographic and clinical characteristics for the cohort are described in Table 1. The average age of individuals at time of testing was 6.6 years, and 60.7% of individuals were male. A majority of individuals (63.1%) had trio GS, with 130 individuals having rapid trio GS and 290 individuals having standard trio GS. Fifty-three (8.0%) individuals had an isolated NDD phenotype.

**Table 1:** Percentage of children with positive GS results across demographic and clinical characteristics of the study cohort.

Cohort characteristic	Sample size *n* (%)	Positive cases *n* (%)
Sex		
Male	404 (60.7%)	114 (28.2%)
Female	262 (39.3%)	93 (35.5%)
Age group		
< 1 year	69 (10.4%)	30 (43.5%)
1 year to < 5 years	259 (38.9%)	78 (30.1%)
5 years to < 18 years	338 (50.8%)	99 (29.3%)
Test type		
Rapid Proband	27 (4.1%)	8 (29.6%)
Rapid Duo	63 (9.5%)	26 (41.3%)
Rapid Trio	130 (19.5%)	63 (48.5%)
Standard Proband	62 (9.3%)	12 (19.4%)
Standard Duo	94 (14.1%)	27 (28.7%)
Standard Trio	290 (43.5%)	71 (24.5%)
Phenotype		
Isolated NDD	53 (8.0%)	5 (9.4%)
Non-Isolated NDD	613 (92.0%)	202 (33.0%)

NDD, neurodevelopmental disorder.

### 3.2 Diagnostic yield of GS for individuals with ID/DD

GS results were positive for 207 individuals (31.1%) with ID/DD. Of the positive cases, 186 had a genetic diagnosis that was at least partially associated with their history of ID/DD, leading to a phenotype-specific diagnostic yield of 27.9% for ID/DD. GS results were indeterminate for 120 individuals (18.0%), and 339 individuals (50.9%) had negative results. Thirty-seven individuals with positive GS results had a previously known genetic diagnosis, with 26 individuals having a genetic diagnosis at least partially related to their clinical presentation of ID/DD (e.g., Trisomy 21 or Klinefelter syndrome), and 11 individuals having a genetic diagnosis that was not related to their clinical presentation of ID/DD (e.g., X-linked ichthyosis or primary hyperoxaluria type 2). Of the individuals with previously known genetic diagnoses, 21.6% (8/37) received at least one additional genetic diagnosis through GS. Eleven cases received positive GS results related to an autosomal recessive (AR) condition that involved the detection of a P/LP variant and VUSP.

The percentage of children with positive GS results was 31.9% (134/420) for trio GS, 33.8% (53/157) for duo GS, and 22.4% (20/89) for proband only GS (χ^2^ (2, *N* = 666) = 3.74, *p* = 0.15). The positive GS rate of rapid GS (44.1%, 97/220) compared to standard GS (24.7%, 110/446) was statistically different (χ^2^ (1, *N* = 666) = 25.96, *p* < 0.001) and was higher in females compared to males (*X*^2^ (1, *N* = 666) = 3.93, *p* < 0.05). The difference in the positive GS rate across the three age groups approached statistical significance (χ^2^ (2, *N* = 666) = 5.57, *p* = 0.06).

The percentage of children with non-isolated NDDs who had positive GS results was 33.0% (202/613) which was significantly higher than the positive GS rate of 9.4% (5/53) for individuals with isolated NDDs (χ^2^ (1, *N* = 666) = 12.60, *p* < 0.001).

Over half (374/666; 56.2%) of individuals had at least one prior genetic test completed that was documented in the clinical note submitted with the testing order, with 87 individuals having at least three genetic tests documented prior to undergoing GS. There were 127 individuals that had ES prior to GS, 227 individuals had a CMA, and 155 individuals had at least one multi-gene panel. For individuals that did not have prior genetic testing or prior testing was not documented, 35.3% (103/292) had positive GS results. For those with at least one prior genetic test, 27.8% (104/374) had positive GS results. The difference in the positive GS rate between individuals with prior genetic testing and those not known to have previous genetic testing was significant (χ^2^ (1, *N* = 666) = 4.27, *p* < 0.05). Specifically, for individuals who previously had ES, 21.3% (27/127) had positive GS. Of those 27 individuals, 40.7% (11/27) had prior positive genetic testing in which GS did not yield new diagnoses, 55.6% (15/27) had one additional genetic diagnosis following GS, and 3.7% (1/27) had one prior genetic diagnosis with GS identifying two additional diagnoses. Notably, there were two individuals with previous ES that received a genetic diagnosis through GS of an AR condition due to a compound heterozygous SNV and CNV, and one individual that was diagnosed with an AR condition due to compound heterozygous pathogenic exonic deletions.

### 3.3 Diverse variants detected by GS

There were 259 variants that led to genetic diagnoses in this cohort. The number and type of variants are summarized in Table 2. Of the 184 nuclear SNVs, 85.3% (157/184) were exonic variants and 14.7% (27/184) were noncoding variants. Of the 27 noncoding variants, 85.2% (23/27) were located 20 or fewer bp from the exon–intron boundary, 11.1% (3/27) were deep intronic variants located > 20 bp from the nearest exon, and one (3.7%) was a variant in *RNU4-2*, a noncoding RNA gene, which is associated with ReNU syndrome (MIM #620851). Eighty-six (46.7%) SNVs were associated with AR conditions, 76 (41.3%) were associated with autosomal dominant (AD) conditions, 15 (8.5%) with X-linked (XL) conditions, and 7 (3.8%) with autosomal dominant and recessive (AD/AR) conditions.

**Table 2:** Variant types involved in genetic diagnoses for individuals with positive GS results.

Variant type	*n* (%)	Coverage of variants on ES+CMA
Mitochondrial genome	2 (0.8%)	No
Complex structural	2 (0.8%)	Limited
STR	4 (1.5%)	No
UPD	1 (0.4%)	Limited
Intronic ≤ 20 bp from exon-intron boundary	23 (8.9%)	Yes
Intronic > 20 bp from exon-intron boundary	3 (1.2%)	Limited
Noncoding transcript variant	1 (0.4%)	No
CNV < 10 kb	7 (2.7%)	Limited
CNV 10 kb – 1 Mb	17 (6.6%)	Yes
CNV > 1 Mb	42 (16.2%)	Yes
Exonic SNV	157 (60.6%)	Yes

STR, short tandem repeat; UPD, uniparental disomy; bp, base pairs; CNV, copy number variant; SNV, single nucleotide variant; CMA, chromosomal microarray analysis; ES, exome sequencing; Coverage is dependent on gene, genomic location, variant, and other factors.

Of the 259 total variants, 25.5% (66/259) were CNVs. Inheritance patterns associated with these CNVs included AD (*n* = 48; 72.7%), AR (*n* = 7; 10.6%), XL (*n* = 10; 15.2%), and Y-linked (*n* = 1; 1.5%). The median size of CNVs that were associated with a genetic diagnosis was 1.7 Mb. The smallest CNV was an approximately 2 kb pathogenic deletion in the *HGSNAT* gene, which was identified in trans with another pathogenic variant, leading to a diagnosis of mucopolysaccharidosis type IIIC (MIM #252930). The largest CNV was a mosaic copy number loss of X chromosome of 152.9 Mb.

Two mitochondrial variants of clinical significance were detected. One individual had a homoplasmic likely pathogenic variant in the *MT-TF* gene, indicating a likely diagnosis of *MT-TF*-related disorders. Another individual had a homoplasmic pathogenic variant in the *MT-TK* gene, consistent with a diagnosis of *MT-TK-*related disorders including Mitochondrial Encephalomyopathy, Lactic Acidosis, and Stroke-like Episodes (MELAS; MIM #540000) and Leigh syndrome (MIM #500017).

Four individuals were identified to have short tandem repeat (STR) expansions. Two individuals had pathogenic expansions in *ATXN2*, consistent with autosomal dominant spinocerebellar ataxia 2 (MIM #183090). One individual had a *DMPK* expansion, consistent with a diagnosis of the congenital form of myotonic dystrophy 1 (MIM #160900). One individual had pathogenic trinucleotide repeat expansions in *ATXN8OS*. Repeat expansions in *ATXN8OS* cause autosomal dominant spinocerebellar ataxia type 8 (MIM #608768).

Two individuals had complex structural variants detected through GS. One individual had a pathogenic paracentric inversion of chromosome 7 with one breakpoint located at intron 6 of the *AUTS2* gene, consistent with a diagnosis of intellectual developmental disorder, autosomal dominant 26 (MIM #615834). The paracentric inversion was previously known through karyotype analysis; however, the clinical consequence was unclear as CMA was normal and the breakpoints of this inversion were not known. The second individual was identified to have a complex rearrangement involving the X chromosome that was predicted to disrupt the gene structure of *NLGN4X* and likely cause X-linked intellectual developmental disorder (MIM #300495).

One individual had maternal uniparental isodisomy of chromosome 14, which is associated with Temple syndrome (MIM #616222).

There were 167 variants related to genetic diagnoses in 134 individuals with positive trio GS results. Of these, 89 variants (53.3%) were considered *de novo* variants, with 76 (85.4%) of the *de novo* variants being associated with AD conditions. Fifty-four *de novo* variants were SNVs (60.7%), 34 were CNVs (38.2%), and one was associated with UPD (1.1%).

### 3.4 GS provides individuals with multiple genetic diagnoses

Of the 207 individuals with positive GS results, 16 individuals had two genetic diagnoses, and one individual had three genetic diagnoses. Eight individuals had one previously known genetic diagnosis, with seven of these individuals being diagnosed with one additional genetic condition through GS and one individual being diagnosed with two additional genetic conditions. Six individuals specifically had an additional diagnosis identified through GS that could at least partially relate to their ID/DD phenotype.

### 3.5 GS also identifies non-diagnostic variants with potential clinical relevance

There were 120 individuals (18.0%) who received an indeterminate GS result, with 164 variants reported in these cases that may have potential relevance to the clinical indication. GS results were indeterminate for 33.7% (30/89) of individuals with proband GS, 19.7% (31/157) of individuals with duo GS, and 14.0% (59/420) of individuals with trio GS. There was a significant association between test type and overall GS result (χ^2^ (4, *N* = 666) = 20.96, *p* < 0.001), driven mainly by a higher frequency of indeterminate results for proband GS and a lower frequency of indeterminate results for trio GS than expected. Twenty-three individuals had a VUSP in a gene associated with an AD condition, and one individual had a VUSP in two different genes that were both associated with an AD condition. A low level (3%) heteroplasmic pathogenic variant in the *MT-TL1* gene was detected in one individual, with the clinical consequence being uncertain due to the low level of heteroplasmy in the blood sample. There were nine individuals in which GS detected an intronic variant that was classified as a variant of uncertain significance, with five being deep intronic variants located > 20 bp from the nearest exon and four being intronic variants located 20 or fewer bp from the exon–intron boundary.

## 4 Discussion

### 4.1 GS as a first-tier test for individuals with ID/DD

This study describes GS results for individuals with ID/DD in one of the largest cohorts to date, enhancing the strength and generalizability of our findings. Results showed that 31.1% of individuals under 18 years of age with a clinical indication of ID/DD had positive GS results. In 27.9% of the total cohort, a genetic diagnosis was identified that was at least partially associated with the clinical history of ID/DD. For individuals without documented genetic testing prior to GS, 35.3% had positive GS results. Both the overall diagnostic yield and phenotype-specific diagnostic yield are comparable to previous studies that show a range in diagnostic yield of GS between 21% and 41% in similar cohorts (14–18).

There were significant differences in the proportion of children with positive GS results across different clinical and test factors. For example, more than 40% of individuals having rapid GS had positive GS results, highlighting the role GS has in obtaining diagnoses for critically ill children with a history of ID/DD. Additionally, more than 40% of children less than 1 year of age had positive GS results, which aligns with a previous study that showed a higher diagnostic yield of ES in children 12–24 months compared to children 25–60 months (19). These differences in yield may be influenced by the age of symptom onset or more severe presentation which led to genetic testing at an earlier age or the need for more rapid testing. The positive GS rate was also significantly higher in females compared to males, with this difference also being noted in previous studies (20, 21).

Although the positive GS rate for trio GS (31.9%, 134/420) and duo GS (33.8%, 53/157) was higher than proband GS (22.4%, 20/89), the difference was not statistically significant (*p* = 0.15). Parental comparators are beneficial in determining inheritance of identified variants which can provide insights into variable expressivity and reduced penetrance of inherited conditions or provide evidence of pathogenicity for *de novo* variants. The proportion of cases with indeterminate GS results were highest for those with proband GS and lowest for those with trio GS, suggesting comparator testing may have additional benefit in reducing uncertain findings. Given that logistical requirements and costs may differ between trio, duo, and proband GS, these findings suggest that access to GS should not be limited by availability of comparator testing.

Results also showed that the proportion of children with positive GS results varied by clinical indication. The positive GS rate for individuals with non-isolated NDDs (33.0%) was higher than the positive GS rate for those with isolated NDDs (9.4%). Differences in diagnostic yields between individuals with isolated and non-isolated NDDs have been shown in previous studies (22). Due to the smaller number of individuals with isolated NDDs in this cohort, as well as limited information regarding phenotype severity, further investigation regarding the diagnostic yield of GS for individuals with isolated NDDs is warranted.

### 4.2 GS reduces the need for multiple genetic tests

The utility of GS as one comprehensive test is demonstrated by multiple findings in this study. First, a key finding was the identification of clinically relevant variants with limited to no detection by conventional ES with CMA. Almost 8% (20/259) of clinically relevant variants identified in this cohort were STRs, complex structural variants, mitochondrial genome variants, deep intronic and noncoding sequencing variants, small CNVs, and UPD, which have limited or no coverage on ES. GS identified at least one new genetic diagnosis in 12.6% (16/127) of individuals with previous ES, with small CNVs identified through GS in several individuals that were not previously reported. GS has the ability to better identify small CNVs, as well as provide more precise breakpoints of structural variants compared to CMA. This was highlighted in the case in which an individual was known to have a paracentric inversion of chromosome 7 of unknown significance due to a normal CMA. GS identified the breakpoint location at intron 6 of the *AUTS2* gene, which was classified as pathogenic. With the ability of GS to detect these multiple variant types in a single test, the need for a stepwise testing approach, which could include karyotype, CMA, mitochondrial genome testing, and repeat expansion testing as individual tests, is reduced.

Second, known genetic diagnoses were confirmed and re-identified through GS, with several individuals having additional genetic diagnoses identified through GS. Having a comprehensive genetic test as a first-tier test allows for the identification of multiple diagnoses at one time and reduces the risk of missing additional genetic diagnoses if further genetic testing is not pursued after the first genetic diagnosis is identified. This is especially valuable for individuals with complex phenotypes that may include multiple, atypical, or unrelated clinical features.

Additionally, over half of individuals had at least one genetic test prior to undergoing GS, with about 20% having ES. While not specifically assessed in this study, there were presumably considerable testing costs, healthcare resources, and time involved in appointments, sample collection, and dissemination of previous genetic testing results. While the cost of GS has historically been more expensive than other genetic tests, GS as a first-tier test may offset costs, as many individuals will not require additional genetic tests (23, 24). However, other factors, including availability of genetics providers, accessibility of GS, and insurance coverage, also influence the use and uptake of GS as a first-tier test.

### 4.3 Potential benefits of multi-omics and emerging technologies following GS

While GS includes a comprehensive analysis of multiple variant types, there are also limitations of GS. Almost one-fifth of individuals received an indeterminate GS result. In some cases, complementary technologies may help clarify pathogenicity and increase diagnostic yield. For example, RNA sequencing can be beneficial for individuals with predicted splicing variants, as can metabolomic testing for individuals with variants in a gene associated with a metabolic pathway. Further, GS has the potential to detect some structural variants as demonstrated by the two individuals in this study in which GS identified complex structural variants; however, certain complex rearrangements, repeat expansions, or highly repetitive genomic regions may be incompletely characterized with short-read sequencing. Optical genome mapping has emerged as a complementary approach that can enhance detection and breakpoint resolution of large or complex structural variants (25). Together, integration of functional and structural genomic technologies may help resolve indeterminate findings and further increase the diagnostic yield beyond GS alone.

### 4.4 Utility of reanalysis of GS data

Given the breadth of GS assays, future reanalysis may be beneficial for those with indeterminate and negative results, as well as for individuals who had positive results that did not fully explain their history of ID/DD. It has been shown that periodic reanalysis increases the yield of ES and GS by 8–21% (26–28). As new gene-disease associations are continuously established and more data is collected which can influence variant classification, individuals may receive a new genetic diagnosis following reanalysis. For example, the individual with ReNU syndrome in this cohort would likely not have received a diagnosis prior to 2024 when this condition was described, but with its description providers can request reanalysis on data from previously performed GS. Future studies regarding reanalysis of GS for individuals with ID/DD could be informative for both clinical and laboratory procedures.

### 4.5 Limitations

This study has several limitations. Phenotypic data, medical records, and previous genetic testing records for each individual in this cohort were limited to the information given by the ordering provider. Individuals could have been excluded from this cohort due to incomplete phenotypic information. For some individuals, the primary indication for GS might not have been an assessment for ID/DD. Additionally, the overall classification of a GS report was considered positive when an identified genetic diagnosis was consistent with any reported clinical phenotype, not only in instances in which the genetic diagnosis fully explained the individual’s ID/DD phenotype. Given the scope of this study, a direct comparison of the diagnostic yield across different genetic tests, such as ES and CMA, was not performed. Additionally, multivariable analyses were not conducted in this study; therefore, all subgroup comparisons should be interpreted cautiously given potential confounding relationships among test and clinical characteristics.

## 5 Conclusion

GS identified at least one genetic diagnosis associated with ID/DD in 27.9% of children with ID/DD as a clinical indication, with an additional 3.2% of children receiving positive GS results associated with other aspects of their clinical indication. Multiple variant types were identified that may not have been detected by other standalone genomic technologies. These findings support the recommendations by professional and medical societies of GS as a first-tier diagnostic test for individuals with ID/DD, as benefits may include shortening the diagnostic odyssey, reducing the number of genetic tests needed for diagnosis, determining presence of multiple genetic diagnoses, and providing more accurate and comprehensive diagnoses for medical management and genetic counseling.

## Data Availability

The raw data supporting the conclusions of this article will be made available by the authors upon reasonable request, without undue reservation.
